# Clinical Translation Challenges and Strategies for Tumour Vaccines Considering Multiple Delivery Routes

**DOI:** 10.3390/vaccines13050469

**Published:** 2025-04-27

**Authors:** Ruiyun Song, Xiao Li, Junsong Zhu, Jian He, Jintong Na

**Affiliations:** 1 State Key Laboratory of Targeting Oncology, Guangxi Medical University, Nanning 530021, China; 2 National Center for International Research of Bio-Targeting Theranostics, Guangxi Medical University, Nanning 530021, China; 3 Guangxi Key Laboratory of Bio-Targeting Theranostics, Guangxi Medical University, Nanning 530021, China; 4 Collaborative Innovation Center for Targeting Tumor Diagnosis and Therapy, Guangxi Medical University, Nanning 530021, China; 5 Guangxi Talent Highland of Major New Drugs Innovation and Development, Guangxi Medical University, Nanning 530021, China

**Keywords:** cancer, tumour vaccine, vaccination modality, injection, microneedle

## Abstract

Background: The high incidence and mortality rates of cancer have kept it at the top of the research agenda for the global healthcare industry, as well as put serious economic pressure on families and society. It has gradually been recognised that reducing the incidence of cancer through various interventions and that combining prevention and treatment are the key to alleviating the burden of cancer. Methods: Retrieve and summarize the literature related to the delivery methods of tumor vaccines, and investigate whether these delivery methods have been applied clinically or have been used in clinical trials. Results: there are a variety of methods for cancer vaccine development, but only a very small number of studies have been able to make strides towards implementing these methods in the clinic, which is closely linked to drawbacks with the means of vaccine delivery. Conclusions: This review analyses the reasons why it is difficult to apply these methods in the clinic from the point of view of the delivery method rather than the design of the cancer vaccine. It also describes some of the delivery methods that have not yet been applied for cancer vaccines and, considering this in conjunction with those that are currently used for this purpose, predicts their prospects for future application.

## 1. Introduction

The latest global cancer statistics published by the International Agency for Research on Cancer (IARC) show that nearly 20 million new cases of cancer occurred in 2022, leading to about 9.7 million deaths, and the number of new cancer cases is expected to reach 35 million by 2050 [1]. Cancer imposes a severe socio-economic burden on countries, not only causing a significant loss of labour and disrupting the balance of the economic structure of many households, but also leading to an increase in healthcare expenditure, with the global economic cost of cancer estimated to be USD 25.2 trillion between 2020 and 2050 [2]. Investments in cancer palliation, treatment, and intervention, as well as in vaccine development, can effectively reduce cancer risks and help tens of thousands of families, with enormous economic benefits and social dividends for countries in the decades to come.

**Figure 1:**
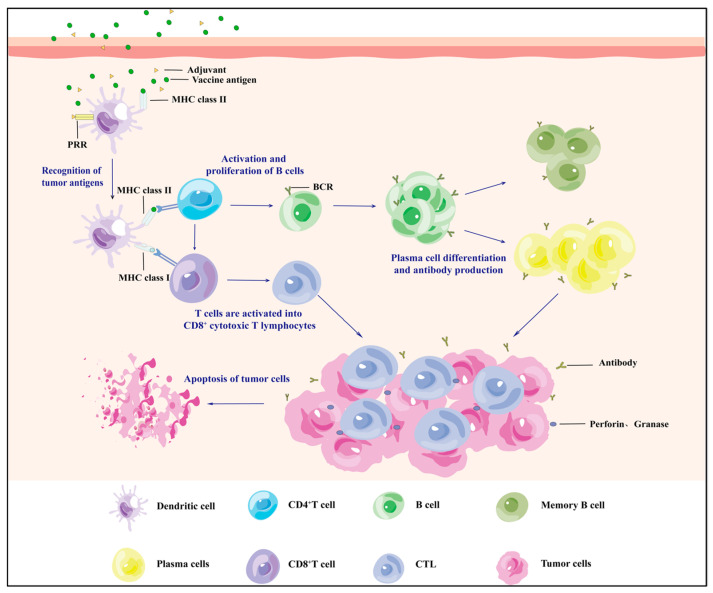
Mechanisms by which tumour vaccines enter the organism to attack tumour cells and induce adaptive immune long-term memory.

Cancer cells can survive in the body by activating a series of signals to evade recognition and clearance by the body’s immune system; thus, finding effective ways to alter the tumour immunosuppressive microenvironment (TEM) is a major challenge in current research. Vaccines are often used in the prevention of infectious diseases, and this approach is also considered to have great potential in attacking the TEM due to a vaccine’s ability to enhance antigen-specific immune responses [3] (Figure 1). Therefore, the focus of cancer prevention and treatment is on the research and development of cancer vaccines. Unlike conventional vaccines, cancer vaccines are vaccines that induce personalised immunity against specific tumours by activating the body’s immune response through the introduction tumour-associated antigens (TAAs) or tumour-specific antigens (TSAs) into the human body to overcome the tumour’s immune evasion function and thus enhance the effectiveness of prevention and treatment methods [4]. Tumour vaccines are divided into two categories according to their uses: therapeutic vaccines and prophylactic vaccines. Therapeutic vaccines are mainly based on tumour antigens and stimulate cytotoxic T cells to produce an effective anti-tumour response. They are usually used in a series of adjuvant treatments after radiotherapy or surgical resection, an example being the world’s first FDA-approved therapeutic tumour vaccine, Provenge (Sipuleucel-T) [5]. Prophylactic vaccines are primarily designed to reduce the incidence or recurrence of tumours by inducing an immune response in the body, and these vaccines are already on the market, such as the HPV vaccine [6], which reduces the incidence of HPV by activating the body’s humoral immunity.

Researchers are constantly screening for effective tumour antigens and have constructed tumour vaccines through a variety of advanced technologies [7]. Cancer vaccines are often based on special materials, such as cells [8], viruses [9], peptides [10,11], and nucleic acids [12]. The preservation and delivery of vaccines has become a concern with regard to maximizing their efficacy, minimizing their cost, and achieving a high vaccination coverage. The safe, efficient, and precise delivery of the carefully developed tumour vaccine to the target site in the body—in order to fully stimulate a strong anti-tumour immune response—has become the next important problem to overcome. This not only affects the efficacy and utilisation of the vaccine, but also serves as a bridge for the application of the vaccine in the clinic.

There are four main types of vaccination methods: nasal spray, inhalation, oral, and injection [13]. Injections have become the most common form of vaccination due to their enhanced effects, but some patients with needle phobias may reject vaccination; thus, some emerging vaccination modalities are now filling this gap. The aim of this review is to summarise the development and advantages of different delivery modes for cancer vaccines; to introduce several new vaccination modes in detail; and to discuss the opportunities and challenges that vaccine delivery faces.

## 2. Traditional Delivery of Oncology Vaccines

### 2.1. Nasal Spray for Vaccination

Nasal spray vaccines, as a new non-invasive vaccine, mainly promote a systemic immune response by activating immune cells in the mucosa of the nasal cavity and the respiratory tract. Since these vaccines can only be deposited into the nasopharynx and cannot reach the lungs, this mode of vaccination is usually used in conjunction with New Crown and influenza vaccines [14]. However, there are still risks for pregnant women, asthmatics, and immunocompromised people, and few cancer vaccines are currently being developed that involve this mode of vaccination.

### 2.2. Inhalation Vaccination

Compared to other organs in the body, the lungs exchange gases with the outside air throughout the day and are easily attacked by microorganisms both inside and outside the body. They also cannot regenerate or repair themselves; therefore, in order to increase the utilisation rate of the vaccine in the lungs and to achieve the desired intrapulmonary sedimentation rate, inhaled vaccines have been developed [15]. Inhaled vaccines differ from nasal spray vaccines: they are nebulized into tiny particles, inhaled via the mouth, and deposited in the lungs [16]. Some scholars have applied this vaccination approach to develop lung cancer vaccines with greater precision. Tang Z et al. developed an inhalation-adapted dual-targeting nanoparticle made of cationic lipids and hyaluronic acid. Its targets include therapeutic mRNAs to lung tumour and inflammatory cells. When inhaled, it shows good targeting and transfection, offering a new method for the palliative treatment of lung infections and cancer metastases [17].

### 2.3. Oral Vaccination

About 70% of the body’s immune cells are in the GI tract. Oral vaccines, given through the GI tract, function by activating intestinal lymphoid tissue immunity. They are a simple method that avoids harm to the circulatory system and vessels [18]. Many research teams are aiming to combine oral delivery with tumour vaccines and are focused on developing oral tumour vaccines to evoke a robust immune response. Lipid nanoparticles (LNPs) are currently a key topic in mRNA delivery [19]. After intravenous injection, mRNA@LNPs may accumulate in the liver, causing adverse reactions. Thus, researchers have introduced the “immune gold” β-glucan to create the oral vaccine β-Glus/mRNA@LNPs. Tests showed that this improved vaccine could shield mRNA from the GI tract’s pH, and once absorbed by intestinal immune cells, it activates T cells, strengthening cancer immunotherapy [20]. Guangjun Nie’s team used genetically modified Escherichia coli outer membrane vesicles (OMVs) to develop an oral cancer vaccine. This vaccine can effectively activate tumour-antigen-specific anticancer responses and inhibit tumour growth, and its ability to suppress melanoma lung metastasis in mice was also explored and verified [21].

Japanese researchers have been continuously developing innovative oral cancer vaccines. In the preliminary stage, they proved that B. longum 420 can effectively inhibit tumour growth in mouse models of prostate cancer and bladder cancer expressing WT1. Building on this foundation, they successively prepared several oral vaccines that can effectively fight against tumours in mouse models of bladder cancer, prostate cancer, and renal cell carcinoma. This has greatly helped patients who are resistant to immune checkpoint inhibitors (ICIs), providing them with a more promising treatment approach [22].

Oral cancer vaccines, being self-administrable, cut costs and have an edge over nasal spray and inhaled vaccines. However, their development has encountered problems. The GI tract’s structure demands that oral vaccines are able to adapt to an acidic pH and resist protein-hydrolysing enzymes. In addition, much higher antigen doses are required for oral vaccines than parenteral ones, risking high-level tolerance in the body [23].

### 2.4. Vaccination by Routine Injection

The injection method, as the most widely used drug delivery method in the medical industry, can not only be used to strictly control the dosage of the drug, but also avoids the impacts that the gastrointestinal tract has on the drug [24]. The site of drug administration is usually determined according to the drug preparation and is generally categorized as intramuscular, subcutaneous, intravenous, or intradermal injection.

#### 2.4.1. Intramuscular Injection

Intramuscular injection is performed using a sterile syringe that allows the drug to be absorbed through the blood vessels in the muscle and into the blood circulation to exert its effect. The most classic example is the HPV vaccine, which has been officially approved for market [25]. The HPV prophylactic vaccine elicits humoral immunity, generating neutralising antibodies to block infection [26]. In addition to this, experts and scholars are improving cancer vaccines to achieve anti-tumour effects efficiently after intramuscular injection. A team at the University of Toronto has successfully designed a new muscle-specific mRNA delivery LNP that can achieve a low off-target rate, and when successfully transported to muscle tissue, can efficiently edit muscle-specific genes. When applied to a mouse melanoma model, after activating the cellular immune response, it was observed to have a strong anti-tumour effect [27].

#### 2.4.2. Hypodermic Injection

The skin is the largest organ in the body and contains a rich capillary network and lymphatic vessels. Skin-mediated drug delivery methods are promising [28]. When a drug is injected subcutaneously, a local drug reservoir forms and the drug enters the systemic circulation via capillary and lymphatic absorption. With benefits such as potential slow release, it is the favoured delivery route for many drugs, particularly macromolecular ones [29]. Professor Hai-Fang Yin’s team studied the efficacy of the subcutaneous injection of neoantigen-loaded exosomes combined with PD-1 inhibitors to treat tumours. They showed that this vaccine boosts tumour immunotherapy, enhances exosome uptake via dendritic cells and lymph node homing, and mobilizes the CD8^+^ T-cell response. Subcutaneous injection of this vaccine significantly curbed tumour growth [30].

In addition to therapeutic tumour vaccines, a team of researchers has developed a subcutaneously administered adjuvant class of red-blood-cell-based vaccines (RBC vaccines) with tumour antigenic modifications targeted to red blood cells to induce long-lasting anti-tumour responses, primarily improving cancer immunotherapy. Subcutaneously injected RBC vaccines can be taken up by DCs, contributing to the increased infiltration of tumour CD8^+^ cells, significantly enhancing the efficacy of αPD-1 therapy, and inducing a shift from CD8^+^ T cells to regulatory T cells. The combined regimen of RBC vaccines and αPD-1 can also induce an expansion of memory T cells, constructing a long-lasting anti-tumour immune response mechanism that attenuates the inhibitory effect of chemotherapy on the immune system, improves the efficacy of chemotherapy, and makes it easier to remove tumour cells [31].

A relatively stable environment is provided for drug absorption via the subcutaneous injection of vaccines, which is conducive to drug action; however, ensuring the effective distribution and long-lasting effect of the drug in specific tissue layers is still a concern for scholars.

#### 2.4.3. Intracutaneous Injection

Through intradermal injection, a drug is delivered from the epidermis to the dermis to ensure local accumulation and drug absorption and action. A CDC research team prepared LMP2 DCs infected with rAd-LMP2. After intradermal vaccination, they found that the vaccine was safe and could effectively prevent nasopharyngeal cancer recurrence and metastasis [32].

Overseas studies have proven the feasibility of intradermal cancer vaccine injection. Sumitomo Pharma America applied for a phase I trial (NCT02498665) in the US to study subcutaneous and intradermal injection of the cancer vaccine DSP-7888 emulsion in advanced cancer patients. Their results showed that intradermal injection was more effective than subcutaneous injection, showing that it had no significant dose-limiting toxicity and better tolerability; thus, intradermal injection was chosen for the next clinical study [33].

Intradermal injection has a great advantage over other injections in terms of the vaccination dose: a very small dose can induce a strong local immune response. However, subcutaneous injection has similar defects to intradermal injection: due to the difficulty of controlling the injection site, it is difficult to accurately control the depth of administration, which affects the immune response, and subcutaneous injection may also cause local adverse reactions.

#### 2.4.4. Intravenous Injection

When tumour vaccines are injected intravenously, as the blood circulates to the immune organs throughout the body, they can recognize and present the antigenic components of the vaccine, thus initiating an immune response [34]. Intravenous injection is a common vaccine delivery method in animal experiments. It allows vaccine antigens to circulate systemically, mimicking tumour cell metastasis in the body. This enables animal immune systems to encounter antigens quickly, which is crucial for studying the immediate immune response and anti-tumour effects of tumour vaccines.

Professor Yongbin Mou’s team explored using nanotechnology to boost dendritic cell (DC)-mediated anti-tumour immune responses by preparing a soft mesoporous organosilica-based nanovaccine (SMONV). After tail-vein injection in mice, the SMONV triggered a strong DC-mediated anti-tumour immune response. It activated tumour-specific T cells, inhibited regulatory T-cell-induced immunosuppression, and sustained the T-cell-mediated immune memory. In melanoma and colon cancer mouse models, the SMONV suppressed tumour growth. When paired with the aPD-1 antibody, it eradicated tumours entirely and established a long-lasting immune memory [35].

Although intravenous injection can rapidly deliver vaccines into the bloodstream for rapid systemic effects, it requires specialized medical personnel to administer the vaccine, which increases healthcare costs and resource requirements; thus, this option needs to be weighed against its associated potential risks.

#### 2.4.5. Intratumoural Injection

The intratumoural injection of tumour vaccines directly acts on tumours, creating high-concentration immune stimulation and triggering a strong immune response. It can also alter the tumour microenvironment, boost immune cell infiltration into tumour sites, and enhance tumour cell death. This method can also be used on distal metastatic tumours, which is crucial for preventing cancer metastasis and recurrence [36].

Researchers from Fudan and the University of Texas developed a small-molecule nanovaccine of PC7A and an antigenic peptide. Its structure allows active delivery to the lymph nodes, priming the immune system. The vaccine activates the STING pathway, is pH-responsive, and promotes antigen cross-presentation, strongly stimulating tumour-specific T cells for anti-tumour effects. Experiments on the subcutaneous and intratumoural injection of this nanovaccine show that, under the same conditions, the latter inhibits tumour growth more efficiently and has a more significant anti-tumour effect [37].

Since most common tumours are not superficial tumours but parenchymal tumours in the body, which makes intratumoural vaccination much more difficult, most of the trials of intratumoural vaccines registered on ClinicalTrials.gov are only for superficial tumours. Only some parenchymal tumours that cannot be surgically removed, or that are difficult and risky to remove surgically, can be treated with intratumoural vaccines as a local treatment to control tumour growth.

All traditional methods of vaccine administration have significant advantages but also disadvantages (Table 1), and these limitations have restricted the vaccine research and development process to a large extent. Most researchers choose to improve the vaccine itself, which undoubtedly leads to huge scientific and technical barriers, while some scientists look for the key to the problem and choose to improve the vaccine’s delivery method to improve its effect and popularity.

## 3. Novel Delivery Modes for Oncology Vaccines

There are problems with traditional tumour vaccination methods: When administered enterally, gastric acid and intestinal proteases can degrade vaccine antigens, reducing immunogenicity and altering the vaccine’s effects. Although intestinal factors do not interfere with parenteral-administered vaccines, the immune response is stable and the dose is controllable, but patient compliance is poor [49]. As a result, researchers are constantly figuring out new ways to vaccinate against tumours to better meet patient needs (Table 2).

### 3.1. Novel Needle-Free Thermal Release-Driven Jet Injector

The new needle-free pyrolysis-driven jet injector (PJI) is based on the principle of the pyrolysis-driven propulsion of the injector and ejects the drug in the form of a high-speed jet from an extremely fine nozzle. This instantly penetrates the skin and enters into the target site, such as subcutaneous tissue or muscle [58]. In order to demonstrate whether the PJI can enable DNA to enter the body through biological barriers such as the skin and achieve good anti-tumour effects, a Japanese research team conducted a study on a transplantable tumour mouse model expressing ovalbumin (OVA) as a model antigen. They found that the protein expression in the skin was higher when using the PJI compared to needle syringes, enhancing antigen-specific CTL production to induce a strong anti-tumour immune response [50].

The PJI can deliver vaccine antigens precisely to the skin’s immune-cell-enriched areas and can also deliver novel vaccines that are difficult to deliver via traditional injection. In addition, its needle-free nature means that it is reusable, avoiding needlestick injury accidents, reducing the occupational risk to healthcare workers, and eliminating the potential for cross-contamination. However, the high-speed drug jet may be painful for the patient and improper operation may cause damage to the skin (leading to certain technical requirements for the operator and professional training). In addition, PJI equipment is expensive, as it requires regular maintenance and calibration, which will increase the cost of healthcare and lead to technical difficulties.

### 3.2. Tumour Vaccine Tattoo

Traditional tattoo artists utilize tattoo needles to uniformly puncture the epidermis at high speeds, implanting dye into the dermis; this retains the design once the wound is closed. Scientists thus came up with the idea that tattoo needles could be used to puncture the skin to stimulate an inflammatory response in the immune system, providing an environment more capable of triggering an immune response for vaccinations [59]. Advances in science and technology have realized the combination of nanotechnology and tattoo technology, completing the expansion of the function of tattoos from purely decorative to medicinal, among other directions.

As early as 2008, German scientists found that the injection of an anti-papillomavirus vaccine into mice through transparent ink tattoos can stimulate an anti-tumour immune response, and a large number of antibodies were produced in the mice. In the same year, a team of researchers conducted a study on melanoma tattoo vaccination and publicly released the results of a phase I clinical trial [60]. Following this, another researcher used the tattoo gun vaccination system to administer pDC-STAMP/OVA to mice to verify that the tattooed vaccine could induce a specific immune response [61]. Scientists have also successively validated the application of tattoo technology to administer various tumour vaccines, such as prostate tumour [62] and human papilloma [63] vaccines, as well observed the therapeutic efficacy and validated the safety of patients with common vulvar intraepithelial neoplasia (uVIN) [53] and melanoma [52].

The development and preparation of tumour vaccine tattoos require highly advanced technology and professional equipment, and the relevant technology is not yet fully mature. The costs of research, development, and production are high, which may limit this technology’s wide application and promotion. This has complicated the research and development of tumour vaccine tattoos, and is also why advances in the field are slow, as the field is relatively small and there are a relatively limited number of scientific researchers who have devoted themselves to it.

### 3.3. Nanogel Delivery of Vaccines

The structure of hydrogels and extracellular matrices is a three-dimensional network that can provide a stable microenvironment for cells and biomolecules. Wrapping a drug in a hydrogel can realise the slow release of the drug to achieve highly efficient medication [64]. The application of hydrogels in tumour vaccine delivery draws on this idea by encapsulating tumour vaccine antigens in a hydrogel to achieve the slow release of antigens, sustained activation of the immune system, and thus long-term anti-tumour immune effects.

The feasibility of implantable hydrogel tumour vaccines has now been demonstrated. After incomplete surgical resection of mice carrying homozygous lateral abdominal Panc02 tumours, the PancVax vaccine, which has been verified to have anti-tumour effects, was encapsulated in a hyaluronic acid hydrogel and implanted at the resection site. It was found that the application of the PancVax hydrogel resulted in the activation of T cells and the expansion of specific T cells, which positively impacted the immunosuppression induced by postoperative PancVax. This induced immunosuppression effectively prevented tumour recurrence after incomplete resection [65]. Although effective, implantable hydrogel vaccines are applied surgically and wound recovery is also slow, with low patient acceptance.

For this reason, the design of an injectable hydrogel tumour vaccine with better patient acceptance has been explored. Injectable hydrogel tumour vaccines have a wide range of applications and can be designed as single-shot prophylactic tumour vaccines to achieve a preventive effect [66]. They can also be used as a multifunctional delivery platform to deliver drugs for the treatment of rectal cancer [67], and vaccines prepared using this system also provide a new solution for preventing recurrence after the surgical resection of melanoma [68]. Hydrogel microspheres [69] obtained by microfluidic device fabrication combine the advantages of nanomaterials and hydrogels [70], removing the need for incisions (as required for implantable hydrogels) and reducing the side effects resulting from the interference of the gelation process after injecting the hydrogel into the organism.

### 3.4. Microneedle Load Tumour Vaccine

As the first line of defence of the human body, the skin consists of the epidermis and the dermis. The outermost layer of the epidermis is the stratum corneum, with multiple layers of flat keratinocytes arranged in close proximity to form a solid ‘wall’ to block the invasion of harmful substances. T-lymphocytes and Langerhans cells (LCs) are important immune cells in the epidermis and play a key role in identifying and removing pathogens and tumour cells in the skin and initiating adaptive immune responses. This is essential for maintaining skin immune homeostasis [71]. Immune cells such as macrophages, mast cells, neutrophils, and dendritic cells in the dermis also make an indelible contribution to the body’s immune defence. Therefore, subcutaneous vaccination is recognised as one of the most effective routes of vaccination.

**Figure 2:**
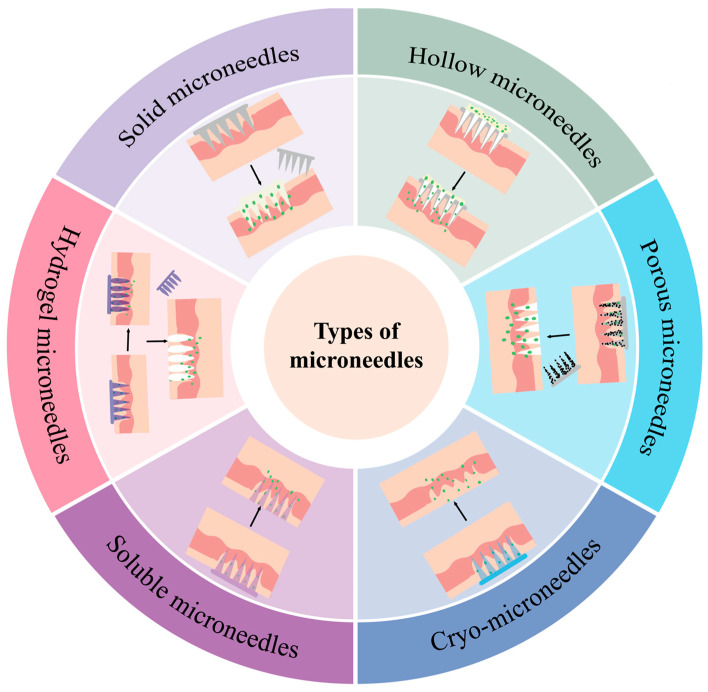
Specifics of different types of microneedle drug delivery.

Microneedling (MN), as a new physical transdermal drug delivery technology, consists of an orderly array of tiny micron-sized tips with a self-contained substrate that can accurately and directionally penetrate the stratum corneum [72], the first line of defence of the skin. Micron-sized fine mechanical channels are opened for vaccine delivery without damaging deep tissues (Figure 2).

The microneedle painlessly penetrates the stratum corneum of the skin and then recruits a large number of DCs and T cells under the skin. After the vaccine (antigen) comes into contact with the DCs in the surface layer of the skin, the DCs will transport the antigen to the lymph nodes and induce an immune response [73]. This stimulates the activation of T cells; thus, the body rapidly produces a large number of CD4^+^ and CD8^+^ T cells to activate its adaptive immune response. A memory response may also be produced when cancer antigens are encountered in the future, thus preventing the occurrence and recurrence of cancer as much as possible. This has the advantages of low cost, high safety, simplicity, painlessness, etc., and it is a minimally invasive and efficient drug delivery method that improves the immunogenicity of the vaccine. This new method of vaccine delivery (Table 3) has shown great potential [74].

In order for microneedles to be able to successfully puncture the skin for drug delivery without affecting their own morphology or that of the drug, a matrix microneedle material is required. Microneedles are usually classified into six categories: solid, hollow, porous, frozen, soluble, or hydrogel. Solid microneedles can deliver tumour vaccines with viruses [75] as the raw material, and their fabrication requires precise techniques and technologies, such as photolithography, etching, and other micromachining technologies. Compared to solid microneedles, hollow microneedles have internal cavities that can hold more drugs, enabling higher drug loading to meet some therapeutic needs. A numerically controlled CNC hollow microneedle injection system developed based on the structure of hollow microneedles (DC-hMN-iSystem) was combined with the cationic liposomal HPV E743-63 SLP vaccine to achieve low-speed vaccine microinjection [77]. This enabled the effective control of tumour growth; however, the construction of this microinjection system has certain technical requirements, which negatively affect its applicability. Porous microneedles, due to the specificity of their structure, are often used as probes to detect changes in organismal biomarkers or indicator changes; scientists have also used porous microneedles to deliver exosomes wrapped with the STING agonist MSA-2 (MEM) [78] as a way to optimize the effect of radiotherapy, effectively preventing the recurrence of tumours. Frozen microneedles have incomparable advantages over other microneedles in the delivery of live cells. Frozen microneedles have been loaded with a live tumour cell vaccine (TCV) and shown, after administration, to recruit more DCs that help control the growth of tumours and improve the quality of the immune response. Frozen microneedles can also significantly increase the infiltration of tumour CD8^+^ T cells, showing great potential in inhibiting melanoma growth and recurrence [79]. Soluble microneedles are widely used in tumour vaccine delivery; one study described the delivery of a cervical cancer vaccine via soluble microneedles, and the inhibition of tumour growth was even better than that achieved by standard intramuscular injections in preclinical models of cervical cancer [82]. Most research on hydrogel microneedles in the field of oncology is linked to photothermal responses or photodynamic therapy [83], but there are many studies proving the feasibility of hydrogel microneedles for delivering exosomes of a cellular origin [84,85,86]; therefore, although there are no systematic studies on the delivery of tumour vaccines via hydrogel microneedles yet, there is huge potential and opportunity lurking.

## 4. Challenges in the Clinical Translation of Oncology Vaccine Delivery Systems

Cancer vaccines are the most promising and cost-effective anti-tumour strategy. In addition to the development of effective anti-tumour vaccines, the selection of the appropriate mode of vaccination also plays a large role in improving the efficiency of the vaccine and reducing the side effects of the drug. The advantages of the injection method, which allow for the administration of large doses and the precise control of the administered dose, have cemented its place in the long history of the research and development of tumour vaccines. However, with the development of the medical industry, people are gradually discovering that high-dose administration also implies an increased risk of allergies and adverse reactions. The emergence of nasal spray vaccines, as well as the development of the PJI, vaccine tattoos, gels, and microneedle arrays, indicate that people are no longer satisfied with the simple and traditional method of vaccination via injection, but are looking for a more efficient and minimally invasive method of delivery.

Compared with the squeeze–push injection of the same vaccine dose, microneedle administration can induce a stronger immune response, and if the matrix of the microneedle is a hydrogel material, it can also achieve the effect of slow release, which enhances the efficacy of the vaccine and reduces its toxicity [87]. In this way, a minimally invasive and painless method is also achieved, which greatly improves the patient’s adherence to medication. In addition, the use of microneedles presents a new direction for administering medication for patients with a ‘needle tip phobia’, which is of great convenience to such special patients. Microneedles with a high biosafety and good compatibility are often made from biodegradable or organic materials, and over time, their structure will change or even the tip of the needle will disappear, thus avoiding the problem of disposal in medical sharp waste [88] and avoiding cross-infection.

Although microneedle vaccination is simple and fast, can be self-administered, and has lower labour costs, in addition to the vaccines being more easily preserved compared to liquid preparations, the cost of production is still a key issue that must be considered. While most microneedle-related research focuses on design and application, the manufacturing of microneedle patches on a large scale is also a central issue in their translation to clinical settings [89]. Few microneedle patches have actually been licensed or entered the medical device market due to the limitations of the manufacturing technology, which cannot support the low-cost mass production of microneedle patches.

## 5. Conclusions and Outlook

Injectable vaccines, oral vaccines, and tumour vaccines prepared as a gel or administered via a microneedle together constitute a multifaceted system of vaccination modes, each with its unique advantages. Microneedle patches are a promising, safer, and less toxic matrix material for tumour vaccine delivery, and have unique benefits.

Compared with other tumour vaccine delivery methods that have entered the clinical research stage, microneedle research is still in the early stages, and there is a significant gap. Although microneedle technology can achieve relatively accurate and efficient drug delivery via vaccines, several key problems still need to be overcome in practical applications.

From the perspective of clinical translation, the first problem is that a standardised production process for microneedle vaccine delivery systems has not yet been realised, which is closely linked to the development of science and technology. Increasing investment in equipment and technology; searching for sterile, durable, safe, and higher-quality manufacturing methods; and increasing the output rate of microneedles via high-quality control will benefit remote areas with poor patient access and achieve cancer prevention in the truest sense.

## Figures and Tables

**Table 1:** Summary of advantages, disadvantages, and applications of traditional injection methods.

Type of Inoculation		Dominance	Shortage	Drug Delivery	Effect	Application	Published
Nasal spray	——	Non-invasive, good patient compliance, faster accumulation from nose to lungs	May cause asthma	SARS-CoV-2 live attenuated vaccine	High safety and efficacy of dNS1-RBD against COVID-19	Clinical trial: ChiCTR2100051391	2023 [38]
				Live attenuated influenza vaccine	Influenza prevention	Data collection and questionnaires	2021 [39]
Inhalation	——	The vaccine is nebulised so that it is inhaled through the mouth and accumulates in the lungs	Not suitable for people with respiratory problems	Negatively charged CAS-LNP formulations	Cancer prevention and treatment vaccine development	Validation in metastatic lung cancer mouse model	2024 [40]
				Dual-target mRNA NPs with cationic lipids and hyaluronic acid	Treatment of lung cancer and pneumonia	Data aggregation and analysis/basic experiments	2024 [41], 2023 [17]
Oral method	——	Vaccines are easy to store and patient compliance is good	Not for those with GI dysfunction; hepatic first pass may reduce vaccine efficacy	βGlus/mRNA@LNPs vaccine	Effective anti-tumour response	Validation on a hormonal mouse model	2024 [20]
				Whole glucan particle WGP-OVA vaccine	Inhibition of tumour growth	Validated in melanoma and LLC mouse tumour models	2022 [42]
Injection	Intramuscular injection	Accurate and wide range of vaccination doses	Not suitable for people with localised skin infections or muscular disorders	LNP-delivered mRNA	Elicited an effective cellular immune response	Validated in a melanoma vaccine model	2023 [27]
	Hypodermic injection	The effects of the vaccine are relatively stable and long-lasting	Limitations on the number of doses of injected vaccines	Neoantigen-coated serum exosomes with PD-1 antibodies	Relieved tumour growth	Melanoma and colon cancer models	2023 [30]
				RBC vaccine	Improvement of chemo-immunotherapy	Colon cancer models	2022 [31]
	Intradermal injection	Good results can be achieved with very small doses	Significant pain and need for professional inoculation	Peptide vaccine rindopepimut	Effective for glioblastoma treatment	Analysis/Clinical Trial: NCT01480479	2022 [43], 2017 [44]
				LMP2-DCs vaccine	Improving NPC patients’ immunotherapy	Patients were followed up	2020 [32]
	Intravenous injection	Accurate dosing enables full vaccine effect	High medical staff skill required, low patient compliance	Pembrolizumab	Treatment of local advanced head–neck SCC	Data Summary and Analysis/Clinical Trial: NCT02641093	2024 [45], 2019 [46]
				mRNA cancer vaccine	Treatment of pancreatic ductal adenocarcinoma	Analysis/clinical trial: NCT04161755	2023 [47], 2023 [48]
	Intratumoural injection	Precise vaccine delivery site reduces drug side effects	Operational errors can cause serious complications	PC7A nanovaccine	Anti-tumour immune response	Studies in TC-1 and B16-OVA cancer models	2022 [37]

**Table 2:** Summary of advantages, disadvantages, and applications of new inoculation methods.

Type of Inoculation	Merits	Shortage	Drug Delivery	Application	Effect	Published
Novel needle-free thermal release-driven jet Injector	Allows precise needle-free drug delivery	Discomfort	OVA expression plasmid DNA	Basic studies using OVA as a model antigen and selection of transplantable tumours expressing OVA E.G7-OVA	Strong anti-tumour immune response	2023 [50]
			DNA vaccine	pOVA as a model antigen and assessment of initial gene expression in the intradermal region	PJI efficiently delivers plasmid DNA to the nucleus in the dermal zone and induces efficient gene expression	2019 [51]
Tattooing	Slow release can be achieved and compliance is good	Immature tech; high R&D and production costs	DNA vaccine (pDERMATT)	Clinical Trial: NCT05309421	New possibilities for cancer vaccine development	2024 [52]
			HPV-16 E6/E7 DNA vaccine	Clinical trial: NTR4607	Good results for uVIN patients	2021 [53]
Nanogel	Immature tech; high R&D and production costs	Harsh storage conditions	Carrageenan gel	Trial registration: ISRCTN96104919	Reduced risk of HPV infection	2021 [54]
			TLR7/8 agonist Resiquimod gel	Clinical Trial: NCT00821652	Effective for resected high-risk melanoma patients	2015 [55]
Microneedle	Easy to operate and no cross-contamination	High cost and lot-to-lot variation	Measles and rubella vaccine	Clinical trial: PACTR202008836432905	May help eliminate measles and rubella	2024 [56]
			Bionic nanovaccine (HAP@Vac)	Validated on a B16 cell-loaded model in C57BL/6 mice	Postoperative treatment of malignant melanoma	2024 [57]

**Table 3:** Design and application of different types of microneedles.

Microneedle Type	Manufacturing Method	Specifications	Matrix Material	Loaded Drug	Application	Published
Solid microneedles	Micromoulding	800 μm height, 200 μm base, and 500 μm pitch	10% PVP and 10% sucrose (20% total concentration)	Adenovirus (PeptiCRAd)	Antitumour effect validation for melanoma and pulmonary carcinoma	2024 [75]
Hollow microneedles	3D printing	Single needle height of 800 μm, bottom diameter of 500 μm	PDMS	——	Detection of glucose and lactate expression levels in ISF	2023 [76]
	Etching	50 μm inner diameter	Sapphire	Cationic liposome HPV E743-63 SLP vaccine	CNC-controlled hollow microneedle injection system for tumour vaccine delivery	2018 [77]
Porous microneedles	Micromoulding	Tip size 250 μm × 250 μm, height 700 μm, MN patch size 1 cm × 1 cm	GelMa	Nano-exosome (EXO)	Optimising the results of FLASH radiotherapy	2024 [78]
Frozen microneedles	Micromoulding	13 × 13 array, 1 mm tip-to-tip distance, tips -900 μm high and 400 μm wide	Culture medium	Live tumour cell vaccine (TCV)	Inhibition of melanoma growth and recurrence	2025 [79]
	Low-temperature micromoulding	Height 950 μm, base width 400 μm	2.5% (*v*/*v*) DMSO and 100 mM sucrose in phosphate-buffered saline (PBS)	Co-delivery of DC vaccine and aPD1	Prevention and treatment of melanoma	2022 [80]
Dissolvable microneedles	Micromoulding	Quadrilateral pyramid-shaped needles, 800 μm high, 300 μm base diameter, in 12 × 12 uniform array, 750 μm tip spacing	Hyaluronic acid (HA) and PVP K90	Antigen-undefined whole tumour cell vaccine (TCV)	Effectively inhibits melanoma invasion and regresses existing malignancies	2023 [81]
	Micromoulding	300 μm diameter and 600 μm length, spaced 50 μm apart	PVA solution	DNA vaccine	Superior to standard intramuscular injection in preclinical cervical cancer models	2018 [82]
Hydrogel microneedles	Two-step process	——	HA	Photothermal agent CCa@TF/Ce6	Combined melanoma treatment and skin regeneration	2024 [83]
	Micromoulding	MNP needle tip spacing is 700 μm in diameter, 500 μm in height, and 270 μm in base	DAM and HAMA	Stem-cell-derived mitochondria-rich EV	Promotes chronic wound healing	2024 [84]

## References

[1] Bray F., Laversanne M., Sung H., Ferlay J., Siegel R.L., Soerjomataram I., Jemal A. (2024). Global cancer statistics 2022: GLOBOCAN estimates of incidence and mortality worldwide for 36 cancers in 185 countries. CA Cancer J. Clin..

[2] Chen S., Cao Z., Prettner K., Kuhn M., Yang J., Jiao L., Wang Z., Li W., Geldsetzer P., Bärnighausen T. (2023). Estimates and Projections of the Global Economic Cost of 29 Cancers in 204 Countries and Territories from 2020 to 2050. JAMA Oncol..

[3] de Visser K.E., Joyce J.A. (2023). The evolving tumor microenvironment: From cancer initiation to metastatic outgrowth. Cancer Cell.

[4] Fan X. (2023). Recent highlights of cancer immunotherapy. Holist. Integr. Oncol..

[5] Anassi E., Ndefo U.A. (2011). Sipuleucel-T (provenge) injection: The first immunotherapy agent (vaccine) for hormone-refractory prostate cancer. Pharm. Ther..

[6] Reuschenbach M., Doorbar J., Del Pino M., Joura E.A., Walker C., Drury R., Rauscher A., Saah A.J. (2023). Prophylactic HPV vaccines in patients with HPV-associated diseases and cancer. Vaccine.

[7] Liu Y., Li S., Chen L., Lin L., Xu C., Qiu H., Li X., Cao H., Liu K. (2024). Global trends in tumor microenvironment-related research on tumor vaccine: A review and bibliometric analysis. Front. Immunol..

[8] Stoitzner P., Romani N., Rademacher C., Probst H.C., Mahnke K. (2022). Antigen targeting to dendritic cells: Still a place in future immunotherapy?. Eur. J. Immunol..

[9] Chen Y., Chen X., Bao W., Liu G., Wei W., Ping Y. (2024). An oncolytic virus-T cell chimera for cancer immunotherapy. Nat. Biotechnol..

[10] Lin X., Tang S., Guo Y., Tang R., Li Z., Pan X., Chen G., Qiu L., Dong X., Zhang L. (2024). Personalized neoantigen vaccine enhances the therapeutic efficacy of bevacizumab and anti-PD-1 antibody in advanced non-small cell lung cancer. Cancer Immunol. Immunother..

[11] Buonaguro L., Tagliamonte M. (2023). Peptide-based vaccine for cancer therapies. Front. Immunol..

[12] Liu Y., Yan Q., Zeng Z., Fan C., Xiong W. (2024). Advances and prospects of mRNA vaccines in cancer immunotherapy. Biochim. Biophys. Acta Rev. Cancer.

[13] Schnyder J.L., Garcia Garrido H.M., De Pijper C.A., Daams J.G., Stijnis C., Goorhuis A., Grobusch M.P. (2021). Comparison of equivalent fractional vaccine doses delivered by intradermal and intramuscular or subcutaneous routes: A systematic review. Travel. Med. Infect. Dis..

[14] Waltz E. (2022). How nasal-spray vaccines could change the pandemic. Nature.

[15] Boboltz A., Kumar S., Duncan G.A. (2023). Inhaled drug delivery for the targeted treatment of asthma. Adv. Drug Deliv. Rev..

[16] Heida R., Hinrichs W.L., Frijlink H.W. (2022). Inhaled vaccine delivery in the combat against respiratory viruses: A 2021 overview of recent developments and implications for COVID-19. Expert. Rev. Vaccines.

[17] Tang Z., You X., Xiao Y., Chen W., Li Y., Huang X., Liu H., Xiao F., Liu C., Koo S. (2023). Inhaled mRNA nanoparticles dual-targeting cancer cells and macrophages in the lung for effective transfection. Proc. Natl. Acad. Sci. USA.

[18] Baker P.J. (2022). Advantages of an Oral Vaccine to Control the COVID-19 Pandemic. Am. J. Med..

[19] Hald Albertsen C., Kulkarni J.A., Witzigmann D., Lind M., Petersson K., Simonsen J.B. (2022). The role of lipid components in lipid nanoparticles for vaccines and gene therapy. Adv. Drug Deliv. Rev..

[20] Luo P.K., Ho H.M., Chiang M.C., Chu L.A., Chuang Y.H., Lyu P.C., Hu I.C., Chang W.A., Peng S.Y., Jayakumar J. (2024). pH-Responsive β-Glucans-Complexed mRNA in LNPs as an Oral Vaccine for Enhancing Cancer Immunotherapy. Adv. Mater..

[21] Yue Y., Xu J., Li Y., Cheng K., Feng Q., Ma X., Ma N., Zhang T., Wang X., Zhao X. (2022). Antigen-bearing outer membrane vesicles as tumour vaccines produced in situ by ingested genetically engineered bacteria. Nat. Biomed. Eng..

[22] Ueki H., Kitagawa K., Kato M., Yanase S., Okamura Y., Bando Y., Hara T., Terakawa T., Furukawa J., Nakano Y. (2023). An oral cancer vaccine using Bifidobacterium vector augments combination of anti-PD-1 and anti-CTLA-4 antibodies in mouse renal cell carcinoma model. Sci. Rep..

[23] Vela Ramirez J.E., Sharpe L.A., Peppas N.A. (2017). Current state and challenges in developing oral vaccines. Adv. Drug Deliv. Rev..

[24] Bitounis D., Jacquinet E., Rogers M.A., Amiji M.M. (2024). Strategies to reduce the risks of mRNA drug and vaccine toxicity. Nat. Rev. Drug Discov..

[25] Carter J.J., Smith R.A., Scherer E.M., Skibinski D.A.G., Sankaranarayanan S., Luxembourg A., Kollmann T., Marty K.D., Sadarangani M., Dobson S. (2025). Term immune memory responses to human papillomavirus (HPV) vaccination following 2 versus 3 doses of HPV vaccine. Vaccine.

[26] Wang M.Y., Cun Y.N. (2023). Current status of clinical trials of HPV therapeutic vaccines. Zhonghua Yu Fang Yi Xue Za Zhi.

[27] Chen J., Xu Y., Zhou M., Xu S., Varley A.J., Golubovic A., Lu R.X.Z., Wang K.C., Yeganeh M., Vosoughi D. (2023). Combinatorial design of ionizable lipid nanoparticles for muscle-selective mRNA delivery with minimized off-target effects. Proc. Natl. Acad. Sci. USA.

[28] Lee D.H., Lim S., Kwak S.S., Kim J. (2024). Advancements in Skin-Mediated Drug Delivery: Mechanisms, Techniques, and Applications. Adv. Healthc. Mater..

[29] Rahimi E., Gomez H., Ardekani A.M. (2022). Transport and distribution of biotherapeutics in different tissue layers after subcutaneous injection. Int. J. Pharm..

[30] Zhang Y., Zuo B., Yu Z., Zhao K., Zhang Y., He K., Seow Y., Yin H. (2023). Complete remission of tumors in mice with neoantigen-painted exosomes and anti-PD-1 therapy. Mol. Ther..

[31] Su L., Hao Y., Li R., Pan W., Ma X., Weng J., Min Y. (2022). Red blood cell-based vaccines for ameliorating cancer chemoimmunotherapy. Acta Biomater..

[32] Zeng Y., Si Y.F., Lan G.P., Wang Z., Zhou L., Tang M.Z., Sj O.B., Lan J., Zhou X.Y., Wang Y.L. (2020). LMP2-DC Vaccine Elicits Specific EBV-LMP2 Response to Effectively Improve Immunotherapy in Patients with Nasopharyngeal Cancer. Biomed. Environ. Sci..

[33] Spira A., Hansen A.R., Harb W.A., Curtis K.K., Koga-Yamakawa E., Origuchi M., Li Z., Ertik B., Shaib W.L. (2021). Multicenter, Open-Label, Phase I Study of DSP-7888 Dosing Emulsion in Patients with Advanced Malignancies. Target. Oncol..

[34] Baharom F., Ramirez-Valdez R.A., Khalilnezhad A., Khalilnezhad S., Dillon M., Hermans D., Fussell S., Tobin K.K.S., Dutertre C.A., Lynn G.M. (2022). Systemic vaccination induces CD8(+) T cells and remodels the tumor microenvironment. Cell.

[35] Li Q., Teng Z., Tao J., Shi W., Yang G., Zhang Y., Su X., Chen L., Xiu W., Yuwen L. (2022). Elastic Nanovaccine Enhances Dendritic Cell-Mediated Tumor Immunotherapy. Small.

[36] Peng S., Tan M., Li Y.D., Cheng M.A., Farmer E., Ferrall L., Gaillard S., Roden R.B.S., Hung C.F., Wu T.C. (2021). PD-1 blockade synergizes with intratumoral vaccination of a therapeutic HPV protein vaccine and elicits regression of tumor in a preclinical model. Cancer Immunol. Immunother..

[37] Jiang X., Wang J., Zheng X., Liu Z., Zhang X., Li Y., Wilhelm J., Cao J., Huang G., Zhang J. (2022). Intratumoral administration of STING-activating nanovaccine enhances T cell immunotherapy. J. Immunother. Cancer.

[38] Zhu F., Huang S., Liu X., Chen Q., Zhuang C., Zhao H., Han J., Jaen A.M., Do T.H., Peter J.G. (2023). Safety and efficacy of the intranasal spray SARS-CoV-2 vaccine dNS1-RBD: A multicentre, randomised, double-blind, placebo-controlled, phase 3 trial. Lancet Respir. Med..

[39] Matsuda K., Migueles S.A., Huang J., Bolkhovitinov L., Stuccio S., Griesman T., Pullano A.A., Kang B.H., Ishida E., Zimmerman M. (2021). A replication-competent adenovirus-vectored influenza vaccine induces durable systemic and mucosal immunity. J. Clin. Investig..

[40] Liu S., Wen Y., Shan X., Ma X., Yang C., Cheng X., Zhao Y., Li J., Mi S., Huo H. (2024). Charge-assisted stabilization of lipid nanoparticles enables inhaled mRNA delivery for mucosal vaccination. Nat. Commun..

[41] Cui S., Chinese Society of Peritoneal Oncology, China Anti-Cancer Association (2023). China Anti-Cancer Association (CACA) guidelines for holistic integrative management of cancer-peritoneal tumours from gastrointestinal tract. Zhonghua Wei Chang Wai Ke Za Zhi.

[42] He L., Bai Y., Xia L., Pan J., Sun X., Zhu Z., Ding J., Qi C., Tang C. (2022). Oral administration of a whole glucan particle (WGP)-based therapeutic cancer vaccine targeting macrophages inhibits tumor growth. Cancer Immunol. Immunother..

[43] Yang T., Shi Y., Liang T., Xing H., Ma W., Li Y.M., Wang Y. (2022). Peptide vaccine against glioblastoma: From bench to bedside. Holist. Integr. Oncol..

[44] Weller M., Butowski N., Tran D.D., Recht L.D., Lim M., Hirte H., Ashby L., Mechtler L., Goldlust S.A., Iwamoto F. (2017). Rindopepimut with temozolomide for patients with newly diagnosed, EGFRvIII-expressing glioblastoma (ACT IV): A randomised, double-blind, international phase 3 trial. Lancet Oncol..

[45] Hou Y.-J., Yang X.-X., Meng H.-X. (2024). Pathological mechanisms and advances in neoadjuvant PD-1 blockade combined with chemotherapy for head and neck cancer. Holist. Integr. Oncol..

[46] Burtness B., Harrington K.J., Greil R., Soulières D., Tahara M., de Castro G., Psyrri A., Basté N., Neupane P., Bratland Å. (2019). Pembrolizumab alone or with chemotherapy versus cetuximab with chemotherapy for recurrent or metastatic squamous cell carcinoma of the head and neck (KEYNOTE-048): A randomised, open-label, phase 3 study. Lancet.

[47] Kang N., Zhang S., Wang Y. (2023). A personalized mRNA vaccine has exhibited potential in the treatment of pancreatic cancer. Holist. Integr. Oncol..

[48] Rojas L.A., Sethna Z., Soares K.C., Olcese C., Pang N., Patterson E., Lihm J., Ceglia N., Guasp P., Chu A. (2023). Personalized RNA neoantigen vaccines stimulate T cells in pancreatic cancer. Nature.

[49] Bouazzaoui A., Abdellatif A.A.H. (2024). Vaccine delivery systems and administration routes: Advanced biotechnological techniques to improve the immunization efficacy. Vaccine X.

[50] Inoue S., Mizoguchi I., Sonoda J., Sakamoto E., Katahira Y., Hasegawa H., Watanabe A., Furusaka Y., Xu M., Yoneto T. (2023). Induction of potent antitumor immunity by intradermal DNA injection using a novel needle-free pyro-drive jet injector. Cancer Sci..

[51] Chang C., Sun J., Hayashi H., Suzuki A., Sakaguchi Y., Miyazaki H., Nishikawa T., Nakagami H., Yamashita K., Kaneda Y. (2019). Stable Immune Response Induced by Intradermal DNA Vaccination by a Novel Needleless Pyro-Drive Jet Injector. AAPS PharmSciTech.

[52] Geukes Foppen M.H., Rohaan M.W., Borgers J.S.W., Philips D., Vyth-Dreese F., Beijnen J.H., Nuijen B., van den Berg J.H., Haanen J. (2024). Intradermal Naked DNA Vaccination by DNA Tattooing for Mounting Tumor-Specific Immunity in Stage IV Melanoma Patients: A Phase I Clinical Trial. Oncol. Res. Treat..

[53] Bakker N.A.M., Rotman J., van Beurden M., Zijlmans H.J.M., van Ruiten M., Samuels S., Nuijen B., Beijnen J., De Visser K., Haanen J. (2021). HPV-16 E6/E7 DNA tattoo vaccination using genetically optimized vaccines elicit clinical and immunological responses in patients with usual vulvar intraepithelial neoplasia (uVIN): A phase I/II clinical trial. J. Immunother. Cancer.

[54] Laurie C., Tota J.E., El-Zein M., Tellier P.P., Coutlée F., Burchell A.N., Franco E.L. (2021). Design and methods for the Carrageenan-gel Against Transmission of Cervical Human papillomavirus (CATCH) study: A randomized controlled trial. Contemp. Clin. Trials.

[55] Sabado R.L., Pavlick A., Gnjatic S., Cruz C.M., Vengco I., Hasan F., Spadaccia M., Darvishian F., Chiriboga L., Holman R.M. (2015). Resiquimod as an immunologic adjuvant for NY-ESO-1 protein vaccination in patients with high-risk melanoma. Cancer Immunol. Res..

[56] Adigweme I., Yisa M., Ooko M., Akpalu E., Bruce A., Donkor S., Jarju L.B., Danso B., Mendy A., Jeffries D. (2024). A measles and rubella vaccine microneedle patch in The Gambia: A phase 1/2, double-blind, double-dummy, randomised, active-controlled, age de-escalation trial. Lancet.

[57] Chen Z., Guo Z., Hu T., Huang B., Zheng Q., Du X., Huang L., Hu W. (2024). Double-layered microneedle patch loaded with bioinspired nano-vaccine for melanoma treatment and wound healing. Int. J. Biol. Macromol..

[58] Sonoda J., Mizoguchi I., Inoue S., Watanabe A., Sekine A., Yamagishi M., Miyakawa S., Yamaguchi N., Horio E., Katahira Y. (2023). A Promising Needle-Free Pyro-Drive Jet Injector for Augmentation of Immunity by Intradermal Injection as a Physical Adjuvant. Int. J. Mol. Sci..

[59] Barber-Axthelm I.M., Kelly H.G., Esterbauer R., Wragg K.M., Gibbon A.M., Lee W.S., Wheatley A.K., Kent S.J., Tan H.X., Juno J.A. (2021). Coformulation with Tattoo Ink for Immunological Assessment of Vaccine Immunogenicity in the Draining Lymph Node. J. Immunol..

[60] Quaak S.G., van den Berg J.H., Toebes M., Schumacher T.N., Haanen J.B., Beijnen J.H., Nuijen B. (2008). GMP production of pDERMATT for vaccination against melanoma in a phase I clinical trial. Eur. J. Pharm. Biopharm..

[61] Moulin V., Morgan M.E., Eleveld-Trancikova D., Haanen J.B., Wielders E., Looman M.W., Janssen R.A., Figdor C.G., Jansen B.J., Adema G.J. (2012). Targeting dendritic cells with antigen via dendritic cell-associated promoters. Cancer Gene Ther..

[62] Babiarova K., Kutinova L., Zurkova K., Krystofova J., Brabcova E., Hainz P., Musil J., Nemeckova S. (2012). Immunization with WT1-derived peptides by tattooing inhibits the growth of TRAMP-C2 prostate tumor in mice. J. Immunother..

[63] van de Wall S., Walczak M., van Rooij N., Hoogeboom B.N., Meijerhof T., Nijman H.W., Daemen T. (2015). Tattoo Delivery of a Semliki Forest Virus-Based Vaccine Encoding Human Papillomavirus E6 and E7. Vaccines.

[64] Ho T.C., Chang C.C., Chan H.P., Chung T.W., Shu C.W., Chuang K.P., Duh T.H., Yang M.H., Tyan Y.C. (2022). Hydrogels: Properties and Applications in Biomedicine. Molecules.

[65] Delitto D., Zabransky D.J., Chen F., Thompson E.D., Zimmerman J.W., Armstrong T.D., Leatherman J.M., Suri R., Lopez-Vidal T.Y., Huff A.L. (2021). Implantation of a neoantigen-targeted hydrogel vaccine prevents recurrence of pancreatic adenocarcinoma after incomplete resection. Oncoimmunology.

[66] Nie X., Shi C., Chen X., Yu C., Jiang Z., Xu G., Lin Y., Tang M., Luan Y. (2023). A single-shot prophylactic tumor vaccine enabled by an injectable biomembrane hydrogel. Acta Biomater..

[67] Yang X., Huang C., Wang H., Yang K., Huang M., Zhang W., Yu Q., Wang H., Zhang L., Zhao Y. (2024). Multifunctional Nanoparticle-Loaded Injectable Alginate Hydrogels with Deep Tumor Penetration for Enhanced Chemo-Immunotherapy of Cancer. ACS Nano.

[68] Liu W.S., Lu Z.M., Pu X.H., Li X.Y., Zhang H.Q., Zhang Z.Z., Zhang X.Y., Shi T., Jiang X.H., Zhou J.S. (2025). A dendritic cell-recruiting, antimicrobial blood clot hydrogel for melanoma recurrence prevention and infected wound management. Biomaterials.

[69] Shao L., Pan B., Hou R., Jin Y., Yao Y. (2022). User-friendly microfluidic manufacturing of hydrogel microspheres with sharp needle. Biofabrication.

[70] Liu J., Du C., Chen H., Huang W., Lei Y. (2024). Nano-Micron Combined Hydrogel Microspheres: Novel Answer for Minimal Invasive Biomedical Applications. Macromol. Rapid Commun..

[71] Zaman R.U., Gala R.P., Bansal A., Bagwe P., D’Souza M.J. (2022). Preclinical evaluation of a microparticle-based transdermal vaccine patch against metastatic breast cancer. Int. J. Pharm..

[72] Faraji Rad Z., Prewett P.D., Davies G.J. (2021). An overview of microneedle applications, materials, and fabrication methods. Beilstein J. Nanotechnol..

[73] Price S.L., Oakes R.S., Gonzalez R.J., Edwards C., Brady A., DeMarco J.K., von Andrian U.H., Jewell C.M., Lawrenz M.B. (2024). Microneedle array delivery of Yersinia pestis recapitulates bubonic plague. iScience.

[74] Babu M.R., Vishwas S., Khursheed R., Harish V., Sravani A.B., Khan F., Alotaibi B., Binshaya A., Disouza J., Kumbhar P.S. (2024). Unravelling the role of microneedles in drug delivery: Principle, perspectives, and practices. Drug Deliv. Transl. Res..

[75] D’Amico C., Fusciello M., Hamdan F., D’Alessio F., Bottega P., Saklauskaite M., Russo S., Cerioni J., Elbadri K., Kemell M. (2025). Transdermal delivery of PeptiCRAd cancer vaccine using microneedle patches. Bioact. Mater..

[76] Cheng J., Huang J., Xiang Q., Dong H. (2023). Hollow microneedle microfluidic paper-based chip for biomolecules rapid sampling and detection in interstitial fluid. Anal. Chim. Acta.

[77] van der Maaden K., Heuts J., Camps M., Pontier M., Terwisscha van Scheltinga A., Jiskoot W., Ossendorp F., Bouwstra J. (2018). Hollow microneedle-mediated micro-injections of a liposomal HPV E7(43–63) synthetic long peptide vaccine for efficient induction of cytotoxic and T-helper responses. J. Control Release.

[78] Chen Z., Hu F., Xiang J., Zhou X., Wu B., Fan B., Tang H., Liu B., Chen L. (2024). Mesoporous Microneedles Enabled Localized Controllable Delivery of Stimulator of Interferon Gene Agonist Nanoexosomes for FLASH Radioimmunotherapy against Breast Cancer. ACS Appl. Mater. Interfaces.

[79] Yang C., Zhao W., Zhang L., He L., Wang S., Wang J., Xiang M., Yuan X., Gou M. (2025). Intradermal Delivery of Cell Vaccine via Ice Microneedles for Cancer Treatment. Adv. Healthc. Mater..

[80] Chang H., Wen X., Li Z., Ling Z., Zheng Y., Xu C. (2023). Co-delivery of dendritic cell vaccine and anti-PD-1 antibody with cryomicroneedles for combinational immunotherapy. Bioeng. Transl. Med..

[81] Yang D., Chen M., Sun Y., Shi C., Wang W., Zhao W., Wen T., Liu T., Fu J., Lu C. (2023). Microneedle-assisted vaccination combined with autophagy regulation for antitumor immunotherapy. J. Control Release.

[82] Cole G., Ali A.A., McCrudden C.M., McBride J.W., McCaffrey J., Robson T., Kett V.L., Dunne N.J., Donnelly R.F., McCarthy H.O. (2018). DNA vaccination for cervical cancer: Strategic optimisation of RALA mediated gene delivery from a biodegradable microneedle system. Eur. J. Pharm. Biopharm..

[83] Zhang Z., Zhang Z., Zeng W., Li Y., Zhu C. (2024). A hyaluronic acid-based dual-functional hydrogel microneedle system for sequential melanoma ablation and skin regeneration. Int. J. Biol. Macromol..

[84] Yao W.D., Zhou J.N., Tang C., Zhang J.L., Chen Z.Y., Li Y., Gong X.J., Qu M.Y., Zeng Q., Jia Y.L. (2024). Hydrogel Microneedle Patches Loaded with Stem Cell Mitochondria-Enriched Microvesicles Boost the Chronic Wound Healing. ACS Nano.

[85] Yuan M., Liu K., Jiang T., Li S., Chen J., Wu Z., Li W., Tan R., Wei W., Yang X. (2022). GelMA/PEGDA microneedles patch loaded with HUVECs-derived exosomes and Tazarotene promote diabetic wound healing. J. Nanobiotechnol..

[86] Han M., Yang H., Lu X., Li Y., Liu Z., Li F., Shang Z., Wang X., Li X., Li J. (2022). Three-Dimensional-Cultured MSC-Derived Exosome-Hydrogel Hybrid Microneedle Array Patch for Spinal Cord Repair. Nano Lett..

[87] Han L., Peng K., Qiu L.Y., Li M., Ruan J.H., He L.L., Yuan Z.X. (2021). Hitchhiking on Controlled-Release Drug Delivery Systems: Opportunities and Challenges for Cancer Vaccines. Front. Pharmacol..

[88] Nguyen H.X., Nguyen C.N. (2023). Microneedle-Mediated Transdermal Delivery of Biopharmaceuticals. Pharmaceutics.

[89] Rai C.I., Kuo T.H., Chen Y.C. (2024). Novel Administration Routes, Delivery Vectors, and Application of Vaccines Based on Biotechnologies: A Review. Vaccines.

